# In-sensor image memorization and encoding via optical neurons for bio-stimulus domain reduction toward visual cognitive processing

**DOI:** 10.1038/s41467-022-32790-3

**Published:** 2022-09-05

**Authors:** Doeon Lee, Minseong Park, Yongmin Baek, Byungjoon Bae, Junseok Heo, Kyusang Lee

**Affiliations:** 1 Department of Electrical and Computer Engineering, University of Virginia, Charlottesville, VA 22904 USA; 2 Department of Electrical and Computer Engineering, Ajou University, Suwon, 16499 South Korea; 3 Department of Materials Science and Engineering, University of Virginia, Charlottesville, VA 22904 USA

**Keywords:** Electrical and electronic engineering, Sensors

## Abstract

As machine vision technology generates large amounts of data from sensors, it requires efficient computational systems for visual cognitive processing. Recently, in-sensor computing systems have emerged as a potential solution for reducing unnecessary data transfer and realizing fast and energy-efficient visual cognitive processing. However, they still lack the capability to process stored images directly within the sensor. Here, we demonstrate a heterogeneously integrated 1-photodiode and 1 memristor (1P-1R) crossbar for in-sensor visual cognitive processing, emulating a mammalian image encoding process to extract features from the input images. Unlike other neuromorphic vision processes, the trained weight values are applied as an input voltage to the image-saved crossbar array instead of storing the weight value in the memristors, realizing the in-sensor computing paradigm. We believe the heterogeneously integrated in-sensor computing platform provides an advanced architecture for real-time and data-intensive machine-vision applications via bio-stimulus domain reduction.

## Abstract (web summary)

Designing in-sensor computing systems remains a challenge. Here, the authors demonstrate artificial optical neurons based on the in-sensor computing architecture that fuses sensory and computing nodes into a single platform capable of reducing data transfer time and energy for encoding and classification.

## Introduction

Machine vision technology provides the capability to inspect and analyze the surrounding environment using image sensors integrated with processing units^1–4^. Recent advances in image sensing and artificial intelligence (AI) technology have enabled the efficient generation of digital images from the physical world and interpretation of the acquired images, respectively. A key feature of machine vision is real-time object recognition and/or classification via machine learning using an artificial neural network (ANN), which is inspired by the visual reasoning process of mammals^5^. Therefore, machine vision has been widely employed in applications that require in situ sensing, environment analysis, and interpretation of visual images, such as autonomous vehicles, robotics, and smart production systems^1,6–8^.

In conventional machine vision systems, image sensors are externally connected to memory and processing units by adapting the von Neumann computing architecture. Sensing occurs in the analog domain, which leads to the generation of large amounts of raw data in the image sensor. The large amounts of redundant analog data, including unnecessary background information, must be converted to digital data via an analog-digital-converter (ADC), then transferred to processing units or cloud-based computing systems^6,9^, in which image processing and analysis are conducted for machine vision applications. Thus, this sensory data processing requires energy-hungry long-distance data communication from the sensors to the memories and processing units with a limited data transfer rate^1,6,10^. This massive amounts of data conversion/transportation causes significant issues in terms of energy consumption, delayed response time, and the communication bandwidth, all of which are important for machine vision applications with strict delay and power-consumption requirements. Therefore, this data transportation bottleneck issue in integrated sensor and processor systems should be mitigated for fast and efficient machine vision processes.

In contrast, the human vision system outperforms conventional imagers and machine vision systems, particularly with regard to unstructured image classification and recognition, low latency, and energy efficiency^6^. The human retina detects images with rod and cone photoreceptors and conveys them through the optic nerve to the brain, which then processes the collected information for visual perception. Interestingly, many of the imaging processes begin in a neural circuit within the retina^11–13^. The retina compresses the large volume of visual signals detected by ~100 million photoreceptor cells and then transmits the encoded data to the brain via the optic nerve, without latency or significant energy consumption. This in-retina computation mechanism can be effectively applied to machine vision systems, alleviating the data transportation bottleneck issues at the sensor/processor interface due to the big data acquisition at the sensor nodes, as well as the heavy computation burden and reliance on the post-processor.

Analogous to the neural circuit and photoreceptor cells in the retina, photodetectors in image sensors can be directly integrated with artificial synapses (e.g., oxide memristors, phase change memories), constructing an artificial neural circuit^14–16^. Integrated analog computing units can store image information as resistance states, while also performing computational tasks to implement an ANN for a cognitive algorithm. In this way, so-called “in-sensor computing,” the image information can be processed within the sensors, significantly reducing data movement by processing it at the edge of the system in a similar way to mammalian vision^1,6^. There have been efforts to demonstrate in-sensor computing systems for analog machine vision using optoelectronic memory devices. However, previously demonstrated in-sensor machine vision systems mostly conducted image memorization and pre-processing (e.g., image enhancement) within the sensors^2,3,17–25^. Thus, all the image data still needs to be transferred to a back-end post-processor for high-level image processing, such as feature extraction, image encoding, and image classification. However, minimizing unnecessary data transportation between the sensor, memory, and computation unit is the key feature and ultimate goal of in-sensor computing to achieve an energy-efficient and latency-free sensor/processor system. As such, advancements in-sensor computing systems require the capability to memorize images and directly perform high-level image processing within the sensor.

In this study, we developed an advanced in-sensor computing system with neuromorphic image memorization and encoding capabilities within the pixel for visual cognitive processing, emulating the biological visual processing system of the mammalian retina and biological long-term plasticity. This in-pixel computing system efficiently computes and conveys visual information to minimize data-transportation bottlenecks. In a single sensor, a photodiode is directly integrated with resistive random-access memory (ReRAM) to construct 1-photodiode 1-resistive random-access memory (1P-1R) pixels, where HfO_2_-based ReRAMs are fabricated on InGaAs-based p-i-n photodiodes. First, we fabricated a 1P-1R single pixel with an InGaAs photodetector and HfO_2_-based ReRAM and characterized the electrical and optoelectrical properties for the image memorization and data processing of visual information within the sensor. Subsequently, a 16 × 16 1P-1R crossbar array with an InGaAs photodiode and a HfO_2_-based ReRAM was fabricated and characterized. Subsequently, the imaging of the MNIST handwritten digits was performed, where the visual stimuli of the images were effectively stored in pixels. Subsequently, using the fabricated 1P-1R array, the biomimicking image encoding process was performed by in-memory vector-matrix-multiplication without data transfer. In contrast to typical in-memory computing methods, input image data were stored in ReRAMs and weights were applied to the crossbar array as input voltages, in which 2D-1D vectorization was no longer needed and the size of the crossbar array was significantly reduced. The encoded images were then conveyed to a central processor for image classification. In the in-sensor computing process, the saved images in the sensor array were directly computed via multiply-accumulate (MAC) operation without any data transportation between an external memory and processor. Our process dramatically reduces redundant data movement between the sensor, memory, and processor by performing in-pixel encoding, possibly alleviating data transportation bottlenecks and energy overconsumption.

## Results

**Fig. 1:**
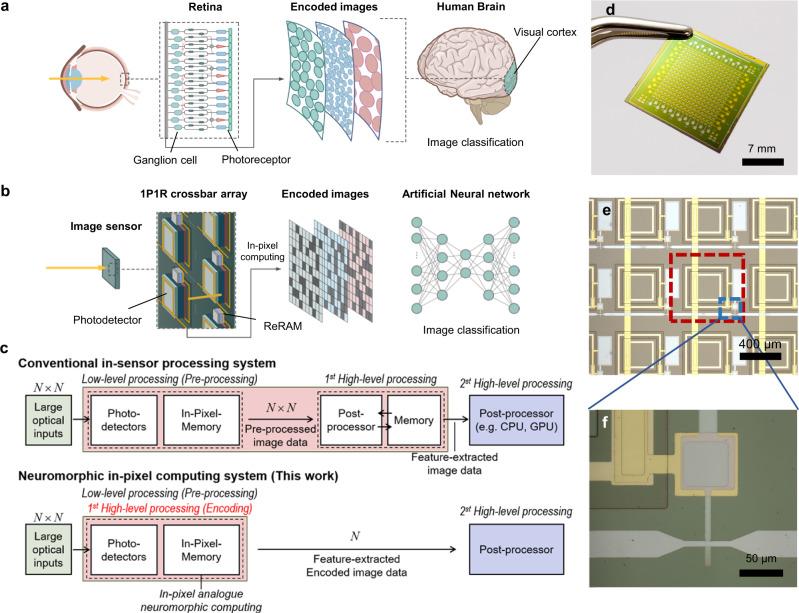
In-sensor computing system with an in-pixel direct computing functionality emulating human vision system. **a** Schematic illustration of human vision system from the retina to the brain, and its visual cognitive processing with in-retina image encoding process. **b** Schematic illustration of a process flow of the 1P-1R in-sensor computing system, which emulates the human vision system. **c** Block diagrams of image process flows with *N*$\times$*N* input images in conventional in-sensor processing systems and 1P-1R in-pixel computing system. **d** Optical image of the fabricated 1P-1R focal plane array. Scale bar: 7 mm. **e** An optical microscope image of the fabricated 1P-1R array. Dashed red (blue) box shows a 1P-1R pixel (ReRAM) in the array. Scale bar: 400 µm. **f** An enlarged optical microscope image of the dashed blue box area in (**e**). Scale bar: 50 µm.

Figure 1a shows a schematic illustration of the image encoding and classification processes in the mammalian retina and brain system. The various types of ganglion cells tile the perceived visual images, and the following bipolar and amacrine cells encode and transmit the information to the brain. The outputs of the rod and cone photoreceptors are decomposed into ~12 parallel information streams, which are then connected to the retinal ganglion cells. Bipolar and amacrine cell activity are combined in a ganglion cell to create diverse encodings of features extracted from the visual world, such as edges, direction, and color; the retina then transmits these pre-processed data to the brain^11–13^. By reducing redundant information, the retina can effectively convey image data to the central processor with a minimal transport delay. In the visual cortex, higher-level visual cognitive processes are conducted using encoded images from the retina^11–13^.

In this study, we designed and demonstrated an in-sensor neuromorphic machine vision system with functionalities of image memorization and processing, by mimicking the above-mentioned neural circuit and visual classification system in the human eye, as shown in Fig. 1b. The image sensor consists of a crossbar array of photodetectors and resistive memory cells, which correspond to photoreceptors and ganglion cells in the retina, respectively. In the retina, the ganglion cells operate as a pre-computing processor unit, whereas the ReRAM in our system serves both as a memory and computation unit, depending on the polarity and magnitude of the applied bias to each pixel. When a reverse bias with respect to the photodiode is applied to the 1P-1R pixels, the sensor operates in a memorization mode where incident light stimuli are converted to electrical signals in the photodetectors, and the photocurrents are subsequently stored in the memory cells by changing the conductance of the memory. Under a forward-biased voltage with respect to the photodiode (lower than the threshold voltage for the erase operation), the sensor operates in the computing mode to process the stored image at the pixels via analog in-memory computing for vector-matrix multiplication. Because vector-matrix multiplication is a key operation in the ANN algorithm, we utilized the 1P-1R crossbar array to execute in-sensor image encoding, which extracts critical features from the original image to alleviate the data transfer burden at the sensor and processor interface, paralleling biological processes in the human retina. Finally, image classification was conducted in the post-processing unit with the encoded images delivered through an ANN. While the encoded images possess compressed information compared to the original images, the ANN successfully classifies the objects with less computational load.

Figure 1c shows block diagrams of the image-processing sequence in conventional in-sensor processing systems and our neuromorphic in-pixel computing system^6,20,22,23^. Most previously reported conventional in-sensor processing systems only perform image memorization and pre-processing (low-level processing) within the sensors, such as image contrast enhancement and noise reduction. Meanwhile, the size of the pre-processed images from the sensors (*N*$\times$*N*) was still the same as the size of the original images (*N*$\times$*N*), and high-level image processing took place in the post-processor. Therefore, conventional systems barely reduce the data traffic load at the sensor/processor interface, as well as the computational burden in the post-processor. In contrast, the fabricated neuromorphic in-pixel computing system in this work memorizes images in each pixel and subsequently conducts image encoding by analog in-memory multiply–accumulate operation (1st high-level processing) by combining the sensing and computing functions. Therefore, the size of the output data from the sensor (*N*) is effectively reduced to the square root of the *N*$\times$*N* original image by the in-sensor image encoding process, thereby minimizing the transportation of redundant data and reducing the computation load in the post-processor. Therefore, this advanced in-sensor computing architecture can significantly reduce the inefficient energy usage and high data latency of smart imaging systems.

**Fig. 2:**
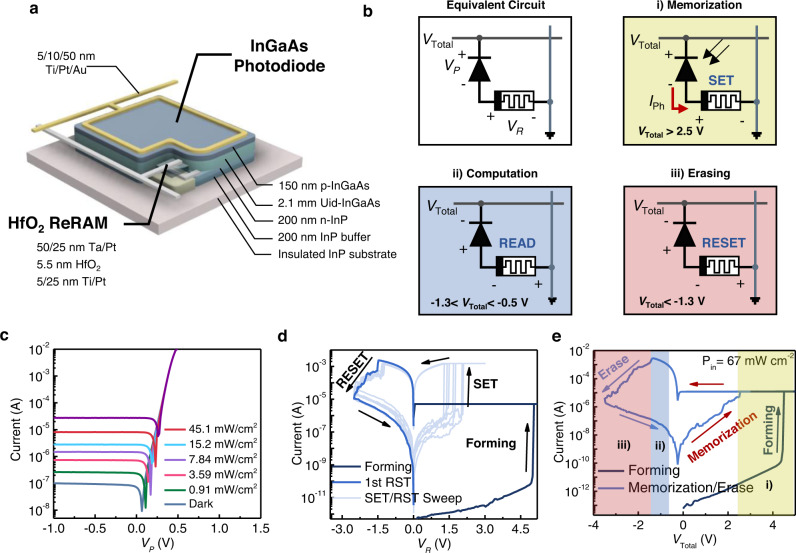
Design and characterizations of single InGaAs 1P-1R integrated device. **a** Schematic illustration of the fabricated InGaAs 1P-1R device. **b** Equivalent circuit diagram of the 1P-1R structure, and three key operations in the 1P-1R device depending on applied bias voltage: (i) memorization, (ii) computation, and (iii) erasing operations. Each background color matches the corresponding operation range colored in (**e**). **c** *I*–*V* curves of the single InGaAs photodiode in the 1P-1R unit under dark and various light illuminations with a wavelength of 532 nm. **d** *I*–*V* characteristic of single ReRAM in the 1P-1R unit, sweeping the applied voltage along with the indicated loops in the graph. **e** *I*–*V* characteristics of the single 1P-1R integrated device under light illumination with an incident power density of 67 mW/cm^2^ and wavelength of 532 nm. The colored regions indicate operation ranges for three main processes shown in (**b**).

First, a 16 × 16 1P-1R in-pixel computing chip was fabricated using p-i-n InGaAs photodiodes, which have a broadband absorption spectrum including the infrared wavelength regime and HfO_2_-based ReRAM (see “Methods”). The optical images of the fabricated chip and optical microscope images of the 1P-1R crossbar array are shown in Fig. 1d and Fig. 1e, f, respectively. Each pixel consisted of an InGaAs photodiode and ReRAM. The row lines share the top electrodes of the ReRAMs (Ta/Pt electrodes) and the column lines share the top electrode of the InGaAs photodiodes (p+ electrodes). Prior to operating the 1P-1R array, we studied the optical and electrical characteristics of a single 1P-1R pixel. Figure 2a shows a schematic illustration of a single 1P-1R pixel, and its equivalent circuit diagram is shown in Fig. 2b, where *V*_*P*_ and *V*_*R*_ are the applied voltages of the photodiode and ReRAM, respectively, and *V*_total_ = *V*_*P*_ + *V*_*R*_. The 1P-1R pixel is composed of an InGaAs p-i-n photodiode and HfO_2_-based ReRAM, which converts incoming optical signals to electrical signals and memorizes the optical information as its resistance. Additionally, the ReRAM is utilized as an in-memory computation unit when it operates in the computation mode. The three primary operations of the 1P-1R device, depending on the applied bias voltage (*V*_Total_), are depicted in Fig. 2b: (i) memorization, (ii) computation, and (iii) erasing operations. When *V*_Total_ > 2.5 V under light illumination, the photodiode is reverse-biased such that an incident optical signal generates a photocurrent to modulate the resistance of the ReRAM by forming a conductive filament (memorization operation). Thus, the optical signal can be stored in the form of resistance in a synaptic device. The stored image data in the ReRAM can then be directly used for high-level in-sensor processing (computation operation). When −1.3 V < *V*_Total_ < −0.5 V, the photodiode operates under the ohmic regime with relatively low resistance (<50 Ω) compared to a resistance range of the ReRAM (>1 × 10^3^ Ω); in this way, the 1P-1R circuit can be approximated to a single ReRAM circuit. Thus, the 1P-1R crossbar array can be used for synaptic in-memory computing based on Ohm’s and Kirchhoff’s laws^6,10,26^. Therefore, ReRAM serves as a cross-functional device for both the memory unit and processing unit for high-level in-sensory image processing. When a high negative bias voltage over the RESET threshold voltage (*V*_Total_ < −1.3 V) is applied across the 1P-1R device, the memorized data in the ReRAM are erased (Erase operation). These three operations are key functions for realizing neuromorphic in-pixel image processing with a 1P-1R crossbar array.

Figure 2c shows the current–voltage (*I*–*V*) characteristics of the fabricated InGaAs photodiode in the single 1P-1R device under light illumination (*λ* = 532 nm) with various light intensities under bias *V*_*P*_, and the bottom electrode on the n-InP layer is grounded. The photocurrents generated from the photodiode under reverse bias modulated the resistance states of the connected ReRAM depending on the incident light intensity. The fabricated HfO_2_-based ReRAM in the single 1P-1R device was also characterized by applying repetitive positive and negative voltage sweeps for the SET and RESET processes, respectively, whereas the bottom electrode (Ti/Pt) of the ReRAM was grounded (Fig. 2d). Initially, to form a conductive filament, a positive DC voltage sweep was employed by increasing the voltage from 0 to 5 V and the voltage was decreased from 5 V to 0 V with 2 μA of compliance current. After the initial channel forming process, repeated SET/RESET operations are performed by applying the positive and negative sweeps of 3 V (SET) and −2.5 V (RESET), respectively, along the sweep paths indicated in the graph to switch the state of the ReRAM between the high-resistance state (HRS) and low-resistance state (LRS). The *I*–*V* curves show stable bipolar switching behavior with abrupt SET and gradual RESET, which is ideal for binary data storage.

The optoelectronic switching behavior of the integrated 1P-1R pixel was then characterized by an *I*–*V* measurement under light illumination (*λ* = 532 nm and *P* = 67 W/cm^2^), as shown in Fig. 2e. Voltage is applied to the top electrode of the ReRAM (Ta/Pt contact) while the top electrode of the photodiode (p-InGaAs contact) is grounded, as shown in the equivalent circuit diagram in Fig. 2b. The ranges of the three corresponding operations, which are explained in Fig. 2b, depending on the applied bias voltage, are indicated by colored areas in the graph. Unlike the electrical-field-driven switching process of a single ReRAM device, the conductive filament channel of the ReRAM in the 1P-1R pixel is grown by photogenerated current from the connected photodiode (memorization operation), whereas RESET switching is still performed by the application of an electrical field. The conductive filament was formed by the first positive voltage sweep under light illumination, corresponding to the forming loop shown in Fig. 2e. After the forming process, the ReRAM is switched to the OFF state by applying a negative voltage sweep, where the light illumination has no effect on the erase operation (Supplementary Fig. 1). Subsequently, a positive voltage sweep was conducted on the 1P-1R device under light illumination, switching the ReRAM to the ON state and memorizing the light information (see memorization loop in Fig. 2d). Under dark conditions, the ReRAM in the 1P-1R device cannot be switched ON via a positive voltage sweep owing to the lack of sufficient driving current to build the conductive filament (Supplementary Fig. 2), because the current flow is limited by the reverse-biased dark current of the photodiode. This result clearly demonstrates the capability of the 1P-1R device as a binary optoelectronic memory.

**Fig. 3:**
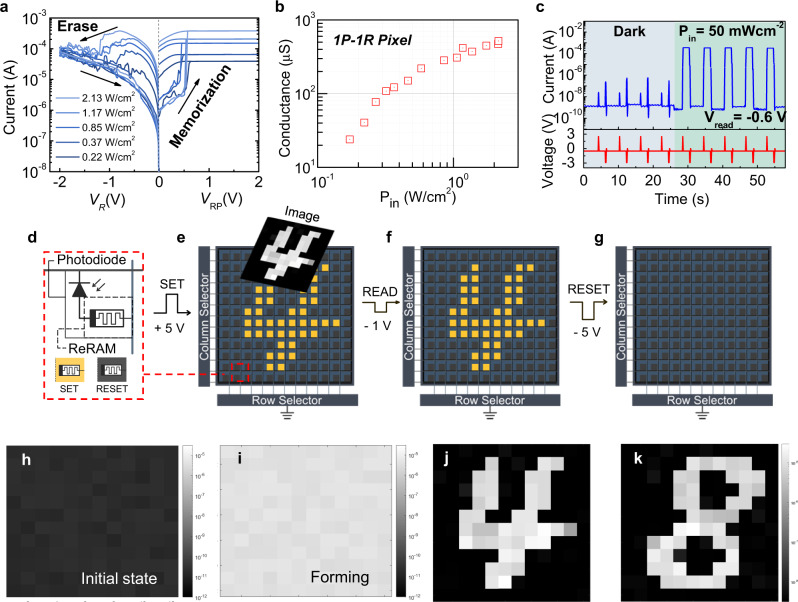
Multi-state optoelectronic memory operation in the single 1P-1R device and characterization and image memorization process of the fabricated 1P-1R crossbar array. **a** Multistate memorization process in the single 1P-1R device under light illuminations with various intensities. Each memorization process is followed by an erase process. **b** Conductance of the ReRAM in the 1P-1R unit as a function of the incident light power density during the memorization process. **c** Sequential memorization process in the single 1P-1R device with voltage pulse inputs. Light with *P*_in_ = 50 mW/cm^2^ is turned on at 26 s. Input voltage pulses of 2 V and −3 V were used for memorization and erase operation, respectively. Otherwise, a voltage of −0.6 V was applied to the device to readout the resistance state of the ReRAM over time. **d** Circuit diagram for a pixel in the 16$\times$16 1P-1R crossbar array. The pixels are colored in yellow (blue) when the ReRAMs are in a SET (RESET) state in (**e**–**g**). Schematic illustration of **e** image memorization, **f** readout, and **g** erasing processes in the 1P-1R crossbar array. Read current maps **h** before and **i** after a forming process in the fabricated 1P-1R array. The read voltage is –1 V. Readout image maps after image memorization of MNIST handwritten number **j** ‘4’ and **k** ‘8’. The read voltage is –1 V.

Moreover, we demonstrate the functionality of the 1P-1R cell as a multistate optoelectronic memory. The memory effect of the 1P-1R unit was predominantly determined by the characteristics of the ReRAM in the pixel. Hence, the multistate capability of the ReRAM enables the 1P-1R pixel to function as a multistate optoelectronic memory device for in-sensor computing applications. Multiple resistance states in the HfO_2_-based ReRAM are usually achieved by the application of various compliance currents during SET operation^27,28^. Supplementary Fig. 3 presents the *I*–*V* curves of the multiple resistance states in the ReRAM by controlling the compliance currents during the SET operations. When a higher compliance current is applied to the ReRAM during the SET operation, a lower resistance state is formed owing to the continuous growth of the oxygen vacancy (*V*_o_)-based filament with a higher charge injection into the oxide layer^27,28^. The achieved multiple conductance states are plotted in Supplementary Fig. 4, depending on the applied compliance. This behavior is consistent with the characterization results from previously reported oxide-based ReRAMs^27–29^. Inspired by the above-described resistance modulation method in HfO_2_-based ReRAMs, the photogenerated currents from the photodiode in the 1P-1R system were used as the driving currents to perform a SET operation on the ReRAM during the memorization process. Therefore, multiple resistance states in the 1P-1R system can be enabled by light illumination with different intensities on the photodiode, generating diverse magnitudes of the driving photocurrent during the memorization process. Figure 3a shows multiple memorization and erase processes to demonstrate the analog resistance states in the 1P-1R device by employing double-voltage sweep *I*–*V* measurements under illumination with 532 nm wavelength light. Positive voltage sweeps were applied across the photodiode-memristor (*V*_RP_) to perform a light-driven SET operation, whereas negative voltage sweeps were applied only to the memristor (*V*_*R*_) for a RESET operation. The *I*–*V* curves in Fig. 3a clearly display the multiple resistance behaviors depending on the incident light intensity, where higher intensity light results in the formation of a filament channel with higher conductance. Figure 3b shows the conductance of multiple states depending on the incident light power density in the memorization process, which is nearly identical to the multistate characteristic of a single ReRAM. However, the nonlinearity of the ReRAM may degrade accuracy in the later image classification task. To obtain linear behavior, one possible approach is to narrow the conductance window so that the focused conductance region can be regarded as a linear region. However, narrowing the conductance window simultaneously decreases the on/off ratio, which also possibly degrades the classification accuracy. Another solution is to employ a logarithmic amplifier that can linearize the exponential transitions. Both approaches have been recently reported^30^ that could further improve the classification accuracy.

The endurance characteristics of the 1P-1R optoelectronic memory were measured after the resistance of the ReRAM was set by illuminating lights with seven different intensities (Supplementary Fig. 5). The measurement results confirm that the 1P-1R device has a stable and reliable endurance property over 10^4 ^s for memory functionality. To validate the continuous image detection and memorization capability using the 1P-1R system, sequential memorization/erase operations are shown in Fig. 3c. Voltage pulses (*V*_RP_) for memorization (2 V), erase (−3 V), and read (−0.6 V) operations were applied to the 1P-1R device under dark or light conditions, with a power density of 50 mW/cm^2^. Without light illumination, no data were stored in the memory even with SET pulses, and only leakage currents were observed. However, the imaging data were stored in the memory once the light illuminated the device with the application of a memorization voltage pulse, and the memory was maintained in the form of an LRS until the application of an erasing voltage pulse. When the erasing voltage pulse was applied to the ReRAM, the device was switched back to the HRS. Repeated memorization/erase processes were successfully completed using the 1P-1R system. This transient switching characteristic can be utilized to perform continuous image memorization/erase processes by applying a voltage pulse train to the 1P-1R focal plane array without data transfer to the external memory. To show the effectiveness of our 1P-1R sensor for fast-switching machine vision applications, we have characterized the optical memorization process with various voltage pulse widths from 10 ns to 100 ms in Supplementary Fig. 6.

Using the fabricated 1P-1R array, we demonstrate in-sensor image storage, encoding, and classification. Prior to image encoding and classification, an image-storage operation was demonstrated. Figure 3d–g shows the schematic illustrations of the image memorization, read, and erasing processes, respectively, with the 16×16 1P-1R focal plane array. A circuit diagram of the pixels is depicted in Fig. 3d, where a ReRAM in a yellow (dark blue)-colored pixel is in the LRS (HRS) state. To control the 1P-1R array, memorization, read, and erase operations (Fig. 2b) were utilized. For the image memorization process, voltage pulses of +5 V (100 µm pulse width) were applied across the individual 1P-1R pixels, where the photodiodes were reverse-biased, to store incident image information in the ReRAMs, as shown in Fig. 3e (see “Methods” for more experimental details). The stored image is then read by applying voltage pulses of −1 V to each 1P-1R pixel, where the photodiode is forward-biased, to read the resistance states of the ReRAMs (Fig. 3f). To erase the saved image in the sensor, voltage pulses of −5 V (100 µm pulse width) were applied to each 1P-1R pixel to switch all pixels to the HRS state, enabling the sensor to be ready to capture the next images (Fig. 3g).

In this study, we characterized and operated a fabricated 16 × 16 1P-1R crossbar array for image memorization under light illumination with a wavelength of 532 nm. First, the *I*–*V* characteristics of an individual pixel in the array were measured under 532 nm light illumination (Supplementary Fig. 7). Forming voltage pulses of +6 V were applied to all 256 pixels under global light illumination (*P* = 70 mW/cm^2^) to form the conductive filament channels in the active medium of ReRAMs. Subsequently, READ voltage pulses of −1 V (100 µm pulse width) were applied to each pixel to read the conductance state of each ReRAM in the pixels, followed by an application of RESET voltage pulses of −5 V to switch all pixels to the HRS state for the next image memorization process. Figure 3h and i shows the 12 × 12 conductance maps of the InGaAs 1P-1R array before and after the forming process, respectively, on a logarithmic scale. Since a 4th row line of the 1P-1R array was damaged during the fabrication process, we have only employed the 12 × 12 array (from 5th to 16th row and column) to obtain images (Supplementary Fig. 8). After the forming process, all pixels were effectively switched from the initial HRS state (Fig. 3h) to the LRS state (Fig. 3i). With the operation-ready 1P-1R array, the memorization function was demonstrated by imaging the handwritten digit images of ‘4’ and 8’ from the MNIST dataset^31^. First, the ‘4’ handwritten digit image is illuminated on the 1P-1R array, and +5 V (SET) voltage pulses are applied to each pixel to memorize the exposed image in the sensor as shown in Fig. 3e (see “Methods” for detailed experimental methods). After the image memorization process, −1 V (READ) voltage pulses were applied to all pixels to read the saved image from the sensor. Figure 3j shows the corresponding current map of the 1P-1R array after the memorization process with the digit ‘4’ image, indicating that the captured image is successfully memorized in the sensor. The saved image in the sensor array is then erased by applying voltage pulses of −5 V (RESET) to all pixels, and second image memorization and readout processes were performed under the image exposure of the MNIST handwritten digit of ‘8’ using the identical procedure described above (Fig. 3k).

The 1P-1R crossbar array can be approximated as a 1R crossbar array under a forward bias condition for the photodiodes because the resistance of the forward-biased p-i-n InGaAs photodiode is relatively low compared to that of the ReRAM. Thus, analog neuromorphic computing can be directly performed in the 1P-1R crossbar array using the stored image data in the ReRAMs in the same way as the 1R-based crossbar arrays^10,16,26,32^. Therefore, image processing and encoding based on ANNs can be conducted within the sensor by directly implementing vector-matrix multiplication. This in-sensor vector-matrix multiplication enables an efficient higher-level computation without data transport between the sensor, memory, and processor, reducing significant amounts of energy consumption and processing time (see Supplementary Table 1, Supplementary Notes 1 and 3)^6,10^.

**Fig. 4:**
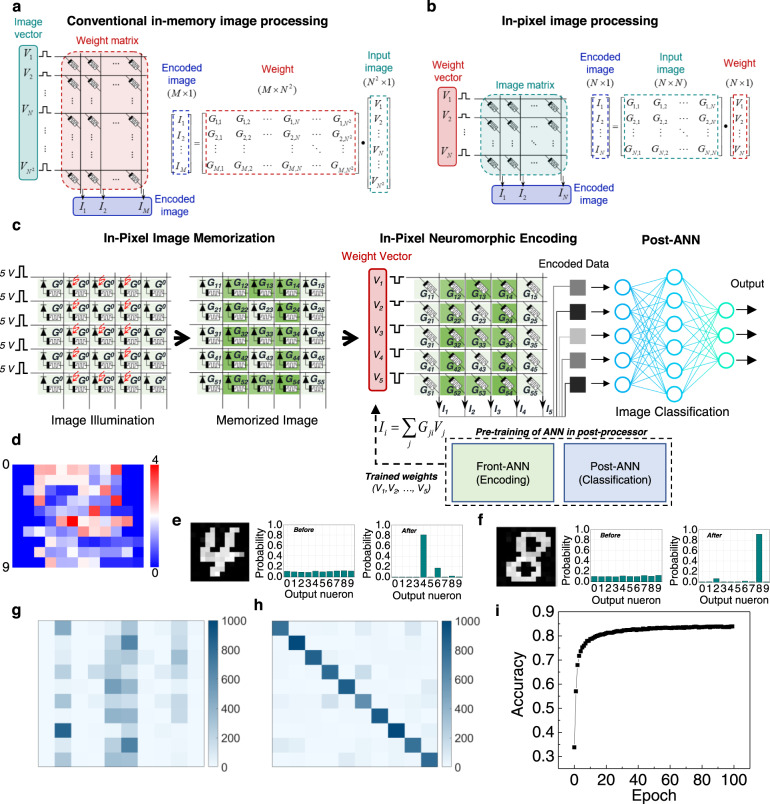
Image memorization, encoding, and classification via an in-pixel neuromorphic computing. Schematic illustration of multiply–accumulate operation for an image encoding process in **a** conventional ReRAM crossbar array and **b** 1P-1R crossbar array. Corresponding matrix-vector multiplication is depicted with parameters of the input voltage (*V*_j_), conductance of ReRAM (*G*_ij_), and output current (*I*_j_). **c** Schematic illustration of an example of in-pixel image memorization, encoding, and classification process with a 5$\times$5 1P-1R array. At the initial state, conductance of ReRAMs is *G*^0^. After image memorization, conductance of each ReRAM is indicated as *G*_ij_. Once an image is memorized in the sensor, pre-trained weight voltages (−1.3 V < *V*_i_ < −0.5 V) are applied to the rows of the crossbar to perform a multiply–accumulate operation in the sensor for the encoding process. The encoded data are transferred to a post-ANN to classify the image. **d** Examples of encoded images of MNIST handwritten numbers from 0 to 9. Classification results from the memorized **e** ‘4’ and **f** ‘8’ digit images be**f**ore and after training the ANN. Confusion matrices for a classification result from the 10,000 MNIST handwritten digit images **g** before and **h** after 100 training epochs. **i** Classification accuracy as a function of the number of training epochs. The classification accuracy is up to 82% with the 100 training epochs.

The fabricated 1P-1R crossbar focal plane array fuses sensing, learning, and computing capabilities similar to those of biological retinas. To realize a neuromorphic vision system, we stored the vision information in each 1P-1R cell as a matrix geometry and simultaneously harnessed the data using emulated vision encoding. Previously demonstrated conventional crossbar geometries of neuromorphic in-memory computing systems for image processing are associated with pre-trained weight values in the ANN matrices, and input image data are applied to the crossbar column as a vectorized electrical signal (Fig. 4a)^6,10,14^. Because the format of image data is usually a 2-dimensional (2D) *N* $\times$ *N* array, 2D-to-1D conversion (vectorization) must be applied as a vector input, which is an *N*^2^ $\times$ 1 vector, to the column of the ReRAM crossbar array. In this case, extra complex circuit components (e.g., ADCs, digital-to-analog converters, and multiplexers) must be added to a peripheral circuit to control a large number of input signals, increasing energy consumption and operational complexity^1,6,10^. However, our in-pixel image processing system transposes image data to the weights of the ANN, in which the input image is applied and stored in the crossbar array in a weight vector matrix form, as shown in Fig. 4b. Therefore, the 2D-to-1D conversion of the image data is no longer necessary for this configuration, significantly reducing the circuit complexity and improving the operational efficiency. Moreover, data transportation from image memorization to the image encoding process is significantly diminished because the image information is directly processed in the pixels without any data transfer.

Figure 4c shows the in-pixel computing process using the fabricated 1P-1R array. The 12 × 12 image of ‘8’ is optically mapped onto the 1P-1R array (sensing) and preserved as the conductance of the ReRAMs (learning). Meanwhile, the front-ANN and post-ANN is pre-trained with 10,000 datasets of the MNIST handwritten numbers in the post-processor to extract the optimum weight vector^31^. The pre-trained 1D weight vector is then converted to electrical signals and applied to the 1P-1R array, enabling the physical matrix multiplication for the in-pixel ANN computation via Ohm’s and Kirchhoff’s laws (computing). The output current signals from the voltage-conductance multiplication thus represent the encoded vector of the image ‘8’, achieved without data transportation by emulating the biological encoding capability. Figure 4d shows the 10 encoded vectors (1 × 12) for the input digits from ‘0’ to ‘9,’ each digit exhibiting a distinguishable encoded vector. The encoded vector is then fed to the next hidden layers to classify the image in the post-ANN (for more details, see Methods). Figure 4e and f shows the classification results from the measured and memorized ‘4’ and ‘8’ digit images. Before training, the activation level of each digit is randomly distributed. However, the activation level of the ANN output neurons of the ANN is concentrated on a single digit after training. The digit with the highest activation level was adopted as the classified ‘answer’. Figure 4g and h shows the results of the image classification before and after 100 training epochs for the full precision of 10,000 test digit images, indicating that the classification performance of the ANN was significantly improved after training the ANN. Although the proposed device has an *N* times smaller number of weight values compared to conventional in-memory computing methods (Fig. 4a), the classification accuracy is up to 82% with 100 training epochs (Fig. 4i). The classification performances are also verified with respect to various SET noises and input bits (Supplementary Figs. 9, 10).

Compared to the general 28 × 28 MNIST handwritten digit dataset, the size of the MNIST dataset employed in this work is much smaller (12 × 12). According to the simulation, increasing the dataset size to the larger pixel (28 × 28) further improves the classification accuracy from 82 to 88.1% (Supplementary Fig. 11 and Supplementary Note 4). Further accuracy improvement could be achieved by employing a dual-encoding neural layer in the ANN, conserving both row- and column-wise features of images, which can be realized by employing bi-directional peripheral circuitry (Supplementary Figs. 12–14 and Supplementary Note 2)^33^. We believe that more practical in-sensor image processing can be realized by increasing the number of pixels in the 1P-1R array integrated with the peripheral circuitry.

We demonstrated a neuromorphic machine vision system with an in-sensor encoding process inspired by mammalian vision. The focal plane array is based on an InGaAs photodiode directly integrated with HfO_2_ ReRAM, constructing the 1P-1R optoelectronic memory and computing pixels. The optoelectronic and memory functionality of the fabricated 1P-1R pixel under light illumination showed reliable digital and multibit memory operation and endurance performance. Furthermore, a 16$\times$16 1P-1R crossbar array with an InGaAs photodiode and HfO_2_-based ReRAM was used to perform edge computing of the handwritten numbers. Finally, we demonstrated biological image encoding with the developed 1P-1R crossbar array, utilizing direct image memorization and in-memory vector matrix multiplication. The encoded images were conveyed to the ANN for image classification, which revealed an accuracy of 82% with 100 training epochs. This slightly lower classification accuracy is attributed to the structure of the encoding neural network, which consists of twelve 12$\times$1 fully connected layers. The architecture of the neural network is inevitably determined by the hardware circuit structure of the 1P-1R crossbar array. The classification accuracy of our sensor system can be further improved by using a dual-encoding neural layer in the ANN. The in-sensor computing concept introduced in this study is a novel method for storing and processing image information directly within pixels without any data transportation between external computing components and is seamlessly scalable with conventional semiconductor fabrication technology.

## Methods

### Device fabrication

InGaAs p-i-n layers were grown on a InP substrate by general molecular beam epitaxy (MBE)^34^. The 1P-1R crossbar array fabrication starts with a mesa etching of p-InGaAs/Uid-InGaAs layers. The mesa areas were protected with a bilayer photoresist (PR; LOR3A/AZ5214) by photolithography, and the unprotected InGaAs area was etched using inductively coupled plasma-reactive ion etching (ICP-RIE; BCl_3_ 20 sccm, 600 W ICP power, 150 W forward power, 7 mTorr, 20 °C stage temperature for 6 min), followed by wet etching for 1 min in a solution of H_3_PO_4_:H_2_O_2_:H_2_O = 3:1:25, which stopped at the n-InP layer. The PR mask was then removed in the Remover PG (Kayaku Advanced Materials) at 60 °C. With a single PR (AZ5214) patterning, n-InP mesa for the bottom metal electrodes was defined, followed by a wet etch process for the n-InP/InP buffer layer using a solution of HCl:H_3_PO_4_ = 3:1 (30 s). Next, a dielectric insulator layer of 150 nm Al_2_O_3_ was deposited by plasma-enhanced atomic layer deposition (PE-ALD). The via holes were etched with a bilayer PR mask using ICP-RIE (BCl_3_ 20 sccm, 50 W ICP power, 200 W forward power, 5 mTorr, 20 °C stage temperature for 6 min). The top and bottom electrodes of the photodiodes were simultaneously deposited by photolithography with bilayer PR and e-beam evaporation of Ti/Pt/Au (5/10/50 nm), which was lifted off in the Remover PG at 60 °C. Another dielectric insulator of 150 nm Al_2_O_3_ was deposited by PE-ALD, and through holes were opened on the bottom electrodes of the photodiodes. Subsequently, the bottom electrodes of the ReRAMs, which are connected to the bottom electrodes of the photodiodes, were deposited using bilayer photolithography, e-beam evaporation of Ti/Pt (5/25 nm), and a lift-off process with the Remover PG. 5.5 nm of a HfO_2_ layer was deposited by PE-ALD, followed by metal deposition of Ta/Pt (50/25 nm) on the top electrodes of the ReRAMs using DC magnetron sputtering of Ta (25 W RF power, 5 mTorr, Ar 20 sccm, room temperature for 18 min) and e-beam evaporation of Pt. Finally, the HfO_2_ mesa areas were defined by dry etching with ICP-RIE with a bilayer PR mask.

### 1P-1R single device characterization

All electrical measurements were performed using a semiconductor analyzer (B1500A, Keysight). The devices were illuminated using a diode laser (DJ532, Thorlabs) with a wavelength of 532 nm, where the incident power was controlled by a neutral density filter.

### Image memorization

MNIST handwritten digit images of ‘4’ and ‘8’ are printed out on photomasks using a direct write lithography tool (MicroWriter ML3, Durham Magneto Optics Ltd), and the diode laser light with a wavelength of 532 nm (*P* = 50 mW/cm^2^) is illuminated on the 1P-1R device by passing through the printed digit images. The projected image (‘4’ or ‘8’) on the sensor is memorized by applying a + 5 V voltage pulse to each pixel using a semiconductor analyzer. After the memorization process, −1 V pulse is applied to each pixel to readout the saved image. Then, the stored image is erased by applying −5 V pulse to each pixel.

### In-pixel image encoding and classification

The encoder and classifier models were implemented using Python. We combined a matrix-to-vector encoder (12 × 12-12) and a fully connected layer classifier with two hidden layers (12-20-16-10) on the Modified National Institute of Standards and Technology (MNIST) dataset. Each original MNIST image was resized to 12 × 12 pixels, and we trained and tested 10,000 images (with 64 batch sizes for 100 epochs) and 100 MNIST images. For the backpropagation learning process, we employed an RMSprop optimizer, rectifier (softmax for the last output) nonlinearity activations, and an initial time-decaying learning rate (0.001).

## Supplementary information

Supplementary Information

## Data Availability

The data supporting the findings of this study are available from the corresponding author upon reasonable request.

## Code availability

All code used in this study is available from the corresponding authors upon reasonable request.
