# Supplementary material for: In-sensor image memorization and encoding via optical neurons for bio-stimulus domain reduction toward visual cognitive processing
Source: Nat Commun. 2022 Sep 5;13:5223. doi: 10.1038/s41467-022-32790-3 (PMC9445171; doi:10.1038/s41467-022-32790-3)
Supplement: Supplementary file 1 — Supplementary Information [file 41467_2022_32790_MOESM1_ESM.pdf]

Supplementary Information for

In-sensor image memorization and encoding via optical neurons for bio-stimulus domain reduction towards visual cognitive processing

Doeon Lee,[1] Minseong Park,[1] Yongmin Baek,[1] Byungjoon Bae,[1] Junseok Heo,[2] Kyusang Lee,[1,3*]*

[1]Department of Electrical and Computer Engineering, University of Virginia, Charlottesville, VA 22904, USA

[2]Department of Electrical and Computer Engineering, Ajou University, Suwon 16499, South Korea

[3]Department of Materials Science and Engineering, University of Virginia, Charlottesville, VA 22904, USA

Keywords: In-sensor computing, neuromorphic computing, image classification, edge-computing, artificial neural network

These authors contributed equally: Doeon Lee, Minseong Park

*e-mail: jsheo@ajou.ac.kr (J.H.), kl6ut@virginia.edu (K.L.)

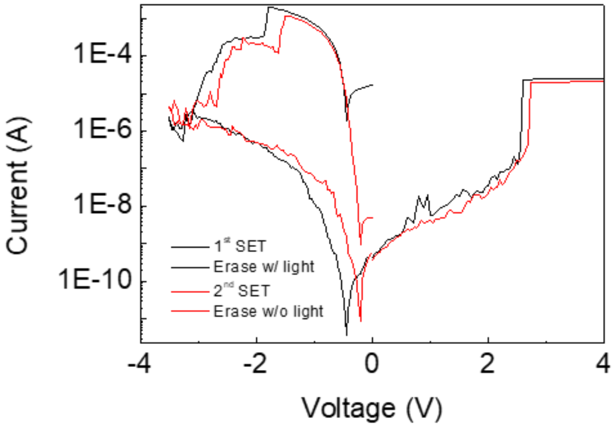

Supplementary Figure 1. I-V curves of erasing process under light illumination and dark condition in the single 1P-1R pixel. The characteristic shows that the ReRAM switches to HRS after erasing process under dark and even light illumination condition.

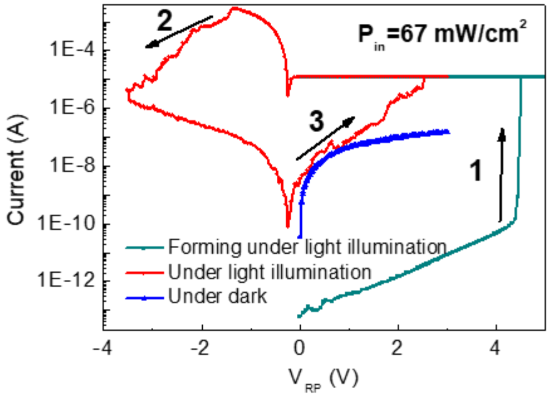

Supplementary Figure 2. I-V characteristic of the single 1P-1R unit from forming to SET and RESET process under light illumination, sweeping the applied voltage along with the indicated loops in the graph. Red (blue) graph shows a positive voltage loop under light illumination (dark) condition.

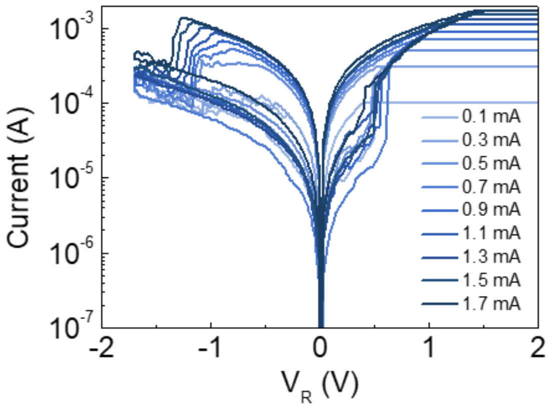

Supplementary Figure 3. I-V characteristics of a single ReRAM in a 1P-1R unit under various compliance currents.

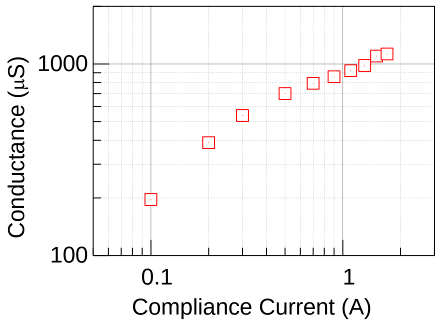

Supplementary Figure 4. Extracted SET conductance from Fig. S3 depending on the compliance current during SET process.

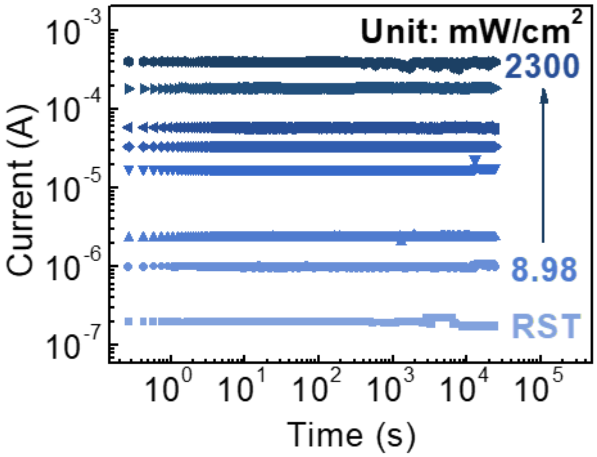

Supplementary Figure 5. Endurance test of 1P-1R optoelectronic memory depending on light intensity during memorization process.

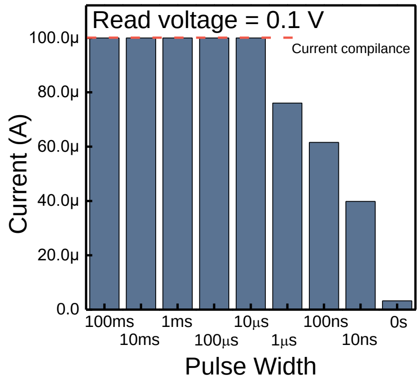

Supplementary Figure 6. Read currents of a 1P-1R pixel after optical memorization with various SET voltage pulse widths (V_{total}=4 V) under light illumination. Although 100-µs voltage pulse width was employed in the manuscript, even a 10-ns SET pulse can also alter the resistance of the ReRAM.

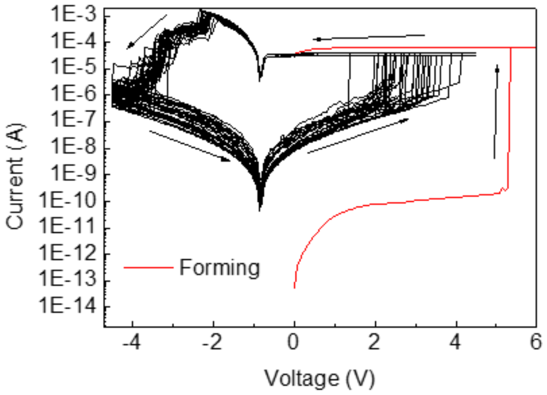

Supplementary Figure 7. I-V characteristics of a 1P-1R pixel in the 1P-1R crossbar array under light illumination. It shows stable switching behavior over repeated memorization/erasing processes.

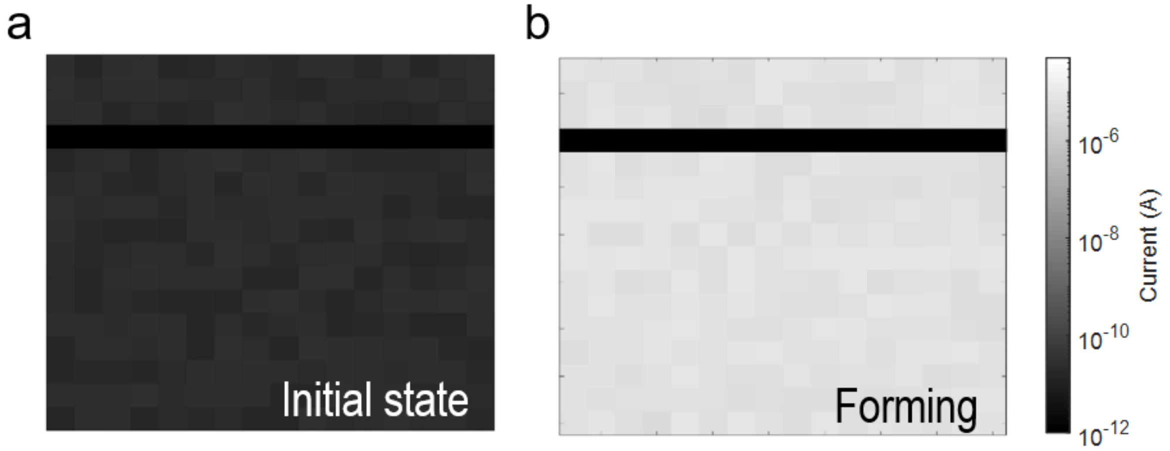

Supplementary Figure 8. Evolution of memristor conductance matrix. **a,b** Read current maps before (**a**) and after (**b**) a forming process in the fabricated 16 × 16 1P-1R array. The read voltage is –1 V

Supplementary Table 1. Comparison of energy/power consumption of single memristor device with recent studies.[1–5]

	Energy	Power
**This work**	**1.275 pJ**	**6.38 nW**
Ref[1]	~ 2fJ	-
Ref[2]	-	1 μW
Ref[3]	6.16 pJ	-
Ref[4]	-	0.6 mW
Ref[5]	~100 fJ	-

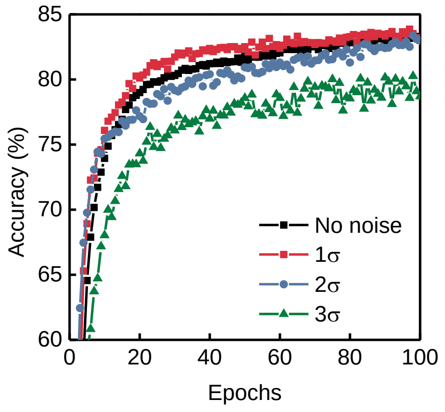

Supplementary Figure 9. Classification accuracy with respect to various "write" noises (σ). The 1σ-noise model results in comparative performance with the no-noise model. Larger noises degraded classification accuracy.

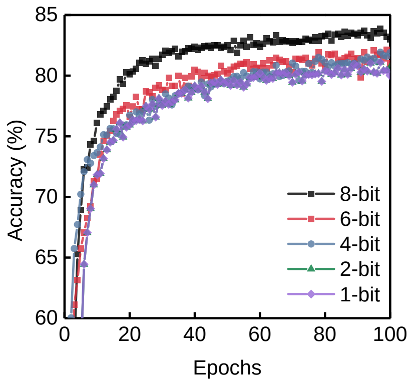

Supplementary Figure 10. Classification accuracy with respect to various input bits. The original 8-bit MNIST dataset is digitized to 1~6-bit dataset. Except the full 8-bit dataset, others perform similar classification accuracy up to approximately 80%. 1σ-noise was applied to all bit cases. Other model parameters are same.

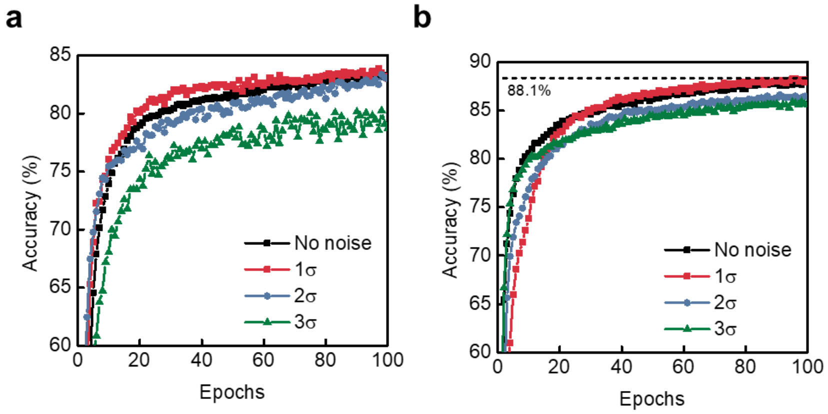

Supplementary Figure 11. Results of MNIST classification. a Classification accuracy with respect to various "write" noises (σ). The 1σ-noise model results in comparative performance with the no-noise model. Larger noises degraded classification accuracy. **b** 28×28 MNIST classification with (28×28)-36-24-10 Encoder + FCN with respect to various "write" noises (σ). Original: (12×12)-20-16-10. Other classification parameters are the same.

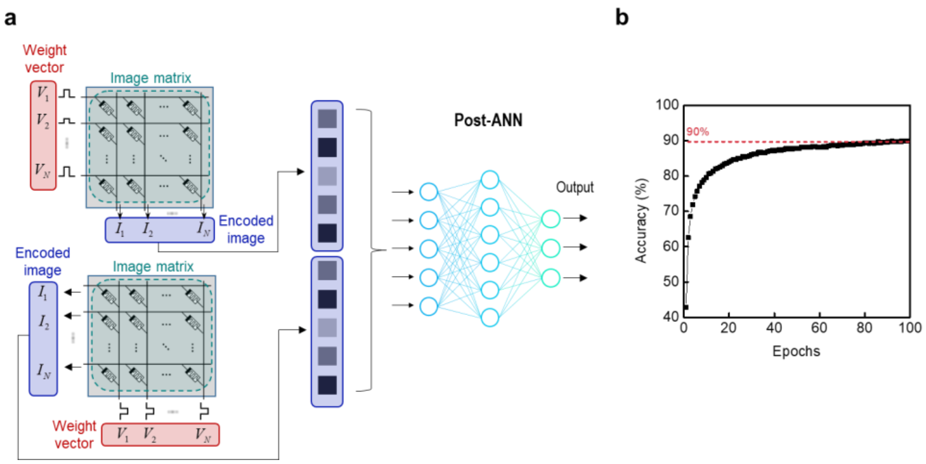

Supplementary Figure 12. Proposed dual-encoding architecture. a Schematic illustration of an example of in-pixel process with dual-encoding architecture. Bi-directional circuitry can realize such dual-encoding capability. **b** 12×12 MNIST classification with dual-encoding scheme. All classification parameters are the same.

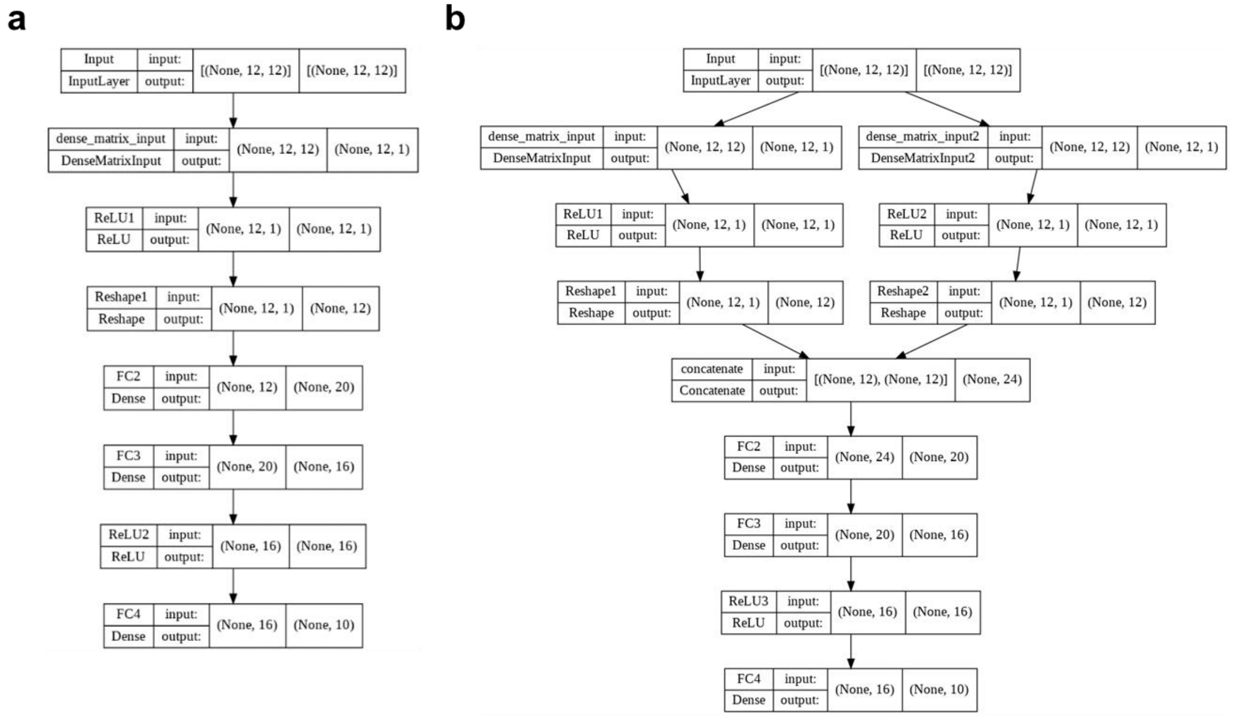

Supplementary Figure 13. 12×12 MNIST classification architectures. a Single-encoding architecture used in this work. **b** Potential dual-encoding architecture for future work.

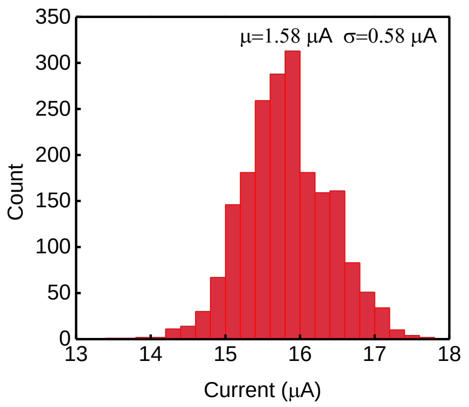

Supplementary Figure 14. Endurance of HfO_2 memristor. The write and erase voltage were 1.4 V and -1.7 V with 1-ms pulse width, respectively. The read voltage was 0.1 V, and the 2000-cycle endurance was characterized.

Supplementary Note 1. Power consumption in the 1P-1R structure

The power consumption of each computing step is calculated for write, read, and erase operations. Based on the results of Fig. 4e, f in the manuscript, the average currents at the high and low resistance states of the memristor are 2.55 nA and 11 μA, respectively. The corresponding pulse amplitudes of write, read, and erase are 5 V, -1 V, and -5 V, respectively. All pulse widths are 100 μs with a 50% duty cycle. Therefore, the power consumption of each process is calculated as follows:

$$P_{\text{write}} = (2.55\ \text{nA}) \times (5\ \text{V}) \times (100\ \mu\text{s}) / (200\ \mu\text{s}) = 6.38\ \text{nW}$$

$$P_{\text{read}} = (11\ \mu\text{A}) \times (1\ \text{V}) \times (100\ \mu\text{s}) / (200\ \mu\text{s}) = 5.5\ \mu\text{W}$$

$$P_{\text{erase}} = (11\ \mu\text{A}) \times (5\ \text{V}) \times (100\ \mu\text{s}) / (200\ \mu\text{s}) = 27.5\ \mu\text{W}$$

$$P_{\text{1cycle}} = P_{\text{write}} + P_{\text{read}} + P_{\text{erase}} + P_{\text{read}} = 38.51\ \mu\text{W}$$

$$P_{\text{1cycle, array}} = (12 \times 12) \times P_{\text{1cycle}} = 5.54\ \text{mW}$$

To the best of our knowledge, the in-pixel encoding 1P-1R architecture has not been demonstrated, thus we alternatively compare the switching power (P_{write}) with other studies (Table S1). However, note that our low-power computing capability is mainly related not to the single memristor operation but to the novel in-sensor computing without reading out all pixels for imaging.

Supplementary Note 2. In-pixel process with dual-encoding architecture

Figure S11 shows the schematic structure of the dual-encoding-based ANN architecture. Once the optical input image is stored in the ReRAM cells, the bi-directional weight column/row vectors allow the dual-encoding functionality. To implement the dual-encoding system, however, bi-directional photocurrent should be generated with bi-directional readout circuitry. Since conventional p-n photodiodes feature only one-directional photocurrent, bi-directional photo-responsive devices can replace such photodiodes, including phototransistors and photoresistors. Note that the original p-n photodiode also functions as a selector, thus the replaced bi-directional optoelectronic devices require an additional transistor as a selector. Therefore, the original fabrication process is replaced with the bidirectional optoelectronic devices in conjunction with the additional transistor fabrication processes. We have previously fabricated the relevant one-transistor one-memristor (1T1R) structure for a neuromorphic application[6].

Although the additional encoding process increases the computing time, the potential dual-encoding system shows an improved classification accuracy (from 82% to 90%) **(**Fig. S12). To clarify the entire artificial neural network architecture (ANN), we also added the flow charts of our original and proposed dual-encoding ANNs **(**Fig. S13**).** The proposed dual-encoding ANN concatenates the row-wise and the column-wise encoded data, and the concatenated data is fed into the PostANN unit as illustrated in Fig. 4c in the manuscript**.** Except for the additional concatenation layer, all the ANN parameters are the same.

Supplementary Note 3. Data bandwidth comparison with conventional CMOS image sensor

The 1P-1R structure stores the image information directly into each “1R” cell, and the resistance is harnessed as an operand of the matrix multiplication without reading the stored data of entire individual pixels (in-pixel computing). Furthermore, the resistive information is stored in a natural analog domain (analog computing), and only single-column ADCs are required to readout the output current (output of the matrix multiplication). To estimate the data bandwidth, we have assumed that the back-end computing time in both approaches is extremely fast (negligeable) compared to data acquisition. Since the proposed in-pixel computing does not require the image data transportation process, we approximate the “frame rate” in the conventional approach to “computing rate”. In particular, the “frame rate” includes times for on/off ratio of photodiodes, readout and reset all pixels. In the case of our approximating “computing rate”, the time includes memorization and encoding of 1P-1R, single-row readout and reset all 1P-1R (Fig. 4c). To simplify the calculation, we assume the identical pulse frequency for all process and compared the data acquisition bandwidth of conventional system with data computation bandwidth of our system[7]. As a result, we have achieved factor of 32.5 (1 OOM) and 250.5 (2 OOM) improved data bandwidth in case of 10k and 1M pixel image sensors, respectively. Array size dependency is mostly due to the single-shot matrix readout process of in-memory computing via MAC operation without data transportation; therefore, it could shorten the readout time dramatically by processing the image at the sensor.

Supplementary Note 4. Classification accuracy depending on noise, the number of pixels, and the number of bits of dataset

We characterized the endurance of the ReRAM by applying a repeated "writing-reading-erasing-reading" scheme to extract noise level of the ReRAMs. As shown in Fig. S14, the standard deviation (σ) of the programming of the ReRAM is 0.58 μA.

Based on the experimentally characterized noise (0.58 μA), we applied the noise to the (encoder + FCN) classification model by converting the current noise to weight noise (the weight values range from 0 to 1). As shown in Fig. S15, the 1σ-noise model exhibits superior classification performance with respect to the no-noise model. However, as the noise level further increases, the performance of the classification models degrades.

Currently, we have fabricated 16×16 array, but the (encoder + FCN) architecture is also scalable when sizing up the dimension of the fabricated array. One critical issue is, however, the limitations of the university fabrication facilities, including misalignment in photolithography, non-uniform distribution of photoresist, and anisotropic etching issues for the scaled structure (more than 16×16, reaching millimetric fabrication processes with micrometric resolution). State-of-the-art industrial nanofabrication facilities can resolve this issue, and the relevant ultra-scaled ReRAMs has been demonstrated for such neuromorphic applications[8,9].

Assuming we can design and fabricate a 1P-1R array with the larger number of pixels as a future work, we possibly improve the accuracy of the image classification based on the proposed in-sensor encoding protocol. Here, we additionally simulated the larger image dataset classification (28×28 MNIST). Following the larger image dimension, we tuned the dimension of the original neural network ((12×12)-20-16-10) to ((28×28)-36-24-10). Other classification

parameters are the same. As shown in Fig. S8, the larger pixel dataset improves the classification accuracy (from 82% to 88.1%).

We also simulated an (encoder + FCN) classifier by employing multi-bit test inputs instead of the original digital input images. Based on Fig. 3b, we assume that the relationship between the illumination intensity and the conductance is approximately linear.

Since the original MNIST dataset is the 8-bit dataset, we digitize the 8-bit dataset to 1-bit, 2-bit, 4-bit, 6-bit datasets in conjunction with the 1σ-noise in Fig. S16. Since the (encoder + FCN) model was trained by the original 8-bit dataset (off-line training in software), the less-bit input images slightly degrade the classification ac curacy, reaching approximately 80%.

Supplementary References

1. Zhang, H. *et al.* Reconfigurable perovskite nickelate electronics for artificial intelligence. *Science* **539**, 533–539 (2022).
2. Wan, F. *et al.* Truly Electroforming-Free Memristor Based on TiO2-CoO Phase-Separated Oxides with Extremely High Uniformity and Low Power Consumption. *Adv. Funct. Mater.* **30**, 1–7 (2020).
3. Xie, Z. *et al.* All-Solid-State Vertical Three-Terminal N-Type Organic Synaptic Devices for Neuromorphic Computing. *Adv. Funct. Mater.* **32**, 1–10 (2022).
4. Jena, A. K. *et al.* Multilevel resistive switching in graphene oxide - multiferroic thin - film - based bilayer RRAM device by interfacial oxygen vacancy engineering. *Appl. Phys. A* **128**, 1–11 (2022).
5. Lu, X. F. *et al.* Exploring Low Power and Ultrafast Memristor on p - Type van der Waals SnS. *Nano Lett.* **21**, 8800–8807 (2021).
6. Lee, H. S. *et al.* Efficient Defect Identification via Oxide MemristiveCrossbar Array Based Morphological Image Processing. *Adv. Intell. Syst.* **3**, 2000202 (2021).
7. Wang, F., Han, L. & Theuwissen, A. J. P. Development and Evaluation of a Highly Linear CMOS Image Sensor With a Digitally Assisted Linearity Calibration. *IEEE J. Solid-State Circuits* **53**, 2970–2981 (2018).
8. Yao, P. *et al.* Fully hardware-implemented memristor convolutional neural network. *Nature* **577**, 641–647 (2020).
9. Kiani, F., Yin, J., Wang, Z., Yang, J. J. & Xia, Q. A fully hardware-based memristive multilayer neural network. *Sci. Adv.* **7**, 1–9 (2021).
